# Racial and Ethnic Differences in Withdrawal of Life-Sustaining Treatment and Hospice Referral in Severe Traumatic Brain Injury

**DOI:** 10.1007/s40615-025-02476-9

**Published:** 2025-05-16

**Authors:** Erin Hennessey, Tamriage Martin, Beth Turrentine, Michelle Pomphrey, Justin Richards, Lliam B. Brannigan, Emily Schneiderman, Bhiken Naik, Michael Mazzeffi

**Affiliations:** 1 Department of Anesthesiology, University of Virginia School of Medicine, Charlottesville, VA USA; 2 Department of Surgery, University of Virginia School of Medicine, Charlottesville, VA USA; 3 Department of Anesthesiology, University of Maryland School of Medicine, Baltimore, MD USA; 4 Department of Anesthesiology, University of Virginia Health, PO Box 800710, Charlottesville, VA 22908 USA

**Keywords:** Trauma, Brain injury, Disparities, Hospice, End-of-life care

## Abstract

**Background:**

Patients with severe traumatic brain injury (TBI) frequently require surrogate decision-making including decisions about end-of-life care. Because prognostication in severe TBI remains challenging, there may be significant variation in surrogate decision-making. Our study aim was to explore differences in withdrawal of life-sustaining treatment (WLST) and hospice referral among patients of different races and ethnicities who had severe TBI.

**Methods:**

A retrospective cohort study was performed using the Trauma Quality Programs Participant Use File from 2022. Patients with severe TBI were identified using diagnostic codes. Severe TBI was defined as Glasgow Coma Scale ≤ 8 at hospital admission. The study’s primary outcomes were WLST and referral to hospice. Multivariable logistic regression models were fit to explore risk-adjusted associations between race and ethnicity, and WLST and hospice referral.

**Results:**

There were 4121 patients included in the final study cohort. Among these, 57.4% were non-Hispanic White, 17.3% were non-Hispanic Black, 14.3% were Hispanic, and 11.0% were non-Hispanic patients of other races. Race and ethnicity were independently associated with WLST. Non-Hispanic Black patients had lower risk-adjusted odds of WLST compared to non-Hispanic White patients; OR = 0.49 (95% CI = 0.37 to 0.65, *p* < .001). Hispanic patients and non-Hispanic patients of other races also had lower risk-adjusted odds of WLST; OR = 0.59 (95% CI = 0.44 to 0.78, *p* < .001) and OR = 0.67 (95% CI = 0.49 to 0.90, *p* = .009). Non-Hispanic Black patients had lower risk-adjusted odds of hospice referral; OR = 0.42 (95% CI = 0.20 to 0.88, *p* = .02) compared to non-Hispanic White patients.

**Conclusions:**

Patients of different races and ethnicities with severe TBI have different rates of WLST and hospice referral. Additional research is needed to better understand the reasons for differences in surrogate decision-making and WLST including the potential influence of culture, religion, socioeconomic status, and medical literacy.

## Introduction

Surrogate decision-making in patients with severe traumatic brain injury (TBI) can be challenging for families, leading to confusion and distress. Patients with severe TBI have a short-term mortality rate between 25 and 45% [1, 2]. Those who survive have a disability rate of up to 50% by 5 years after their injury [3]. Notably, by 5 years, nearly an equal percentage of patients has worsened or improved [3]. By 20 years after initial injury, up to 37% of patients with severe TBI have a good functional recovery, improve to the degree where they are able work, and have comparable neuropsychological testing compared to others in their community [4].

Many factors can impact surrogate decision-making including presence of an advance directive, medical literacy of the surrogate, culture, religion, socioeconomic status, and the direction provided by healthcare professionals. Trauma patients rarely have an advance directive [5, 6], and frequently surrogate decision makers are left to make the best possible decision with the information at hand, along with their knowledge of a patient’s prior wishes, a concept referred to as substitute judgment [7].

Accurate prognostication in severe TBI is fraught with difficulty, and a 2024 guideline described the prediction of functional outcome as “extremely difficult” [8]. Given this uncertainty, families can reasonably make a variety of decisions under the same clinical circumstances based on individual preferences. The decision about whether to terminate or continue life-sustaining treatment, or pursue palliative care, has many practical consequences, as well as potentially irreversible psychological and emotional consequences. These include impacting the timing of death, quality of life, and major financial consequences for individuals and the healthcare system. Multiple prior studies in critically ill patients, including trauma patients, have suggested that there are differences in the withdrawal of life-sustaining treatment (WLST) and hospice referral among patients of different races and ethnicities [9–12].

Differences in end-of-life care among patients of different races and ethnicities with severe TBI have been only sparingly studied [13], and recent trends in both WLST and hospice referral after severe TBI have not been reported to our knowledge. The primary aim of our study was to explore differences in WLST among patients of different races and ethnicities. A secondary aim was to explore differences in hospice referral among patients of different races and ethnicities. We hypothesized that there would be significant differences in both the percentage of patients who had WLST and utilized hospice based on race and ethnicity.

## Methods

### Patients and Patient Data

The School of Medicine’s Institutional Review Board for Health Sciences Research determined that the study was not human subjects’ research and did not require written informed consent. Subjects with TBI were identified in the 2022 Trauma Quality Programs (TQP) Participant Use File (PUF) using International Classification of Disease (ICD)−10 codes, Tenth Revision, Clinical Modification (S061, S062, and S063 families). Patients with severe TBI were defined as those with an initial Glasgow Coma Scale of 8 or less and were included in the study cohort. For all patients, we collected demographics, available medical comorbidities, insurance information, hospital data, initial vital signs, information about the TBI severity, injury severity score (ISS), advanced directive status, transfusion data for the first 4 h, complications during hospitalization, total mechanical ventilation time, intensive care unit (ICU) and hospital length of stay, and discharge information.

### Exposure

In accordance with recent recommendations for disparities research, patient race and ethnicity were categorized as non-Hispanic White, non-Hispanic Black, Hispanic, and non-Hispanic patients of other races [14]. Race and ethnicity data in the TQP PUF are either self-reported or reported by a family member per the database’s dictionary. There are six race categories in the database, which are based on 2010 census categories; however, these were consolidated for the study given sample size considerations.

### Study Outcomes

The study’s primary outcomes were WLST, and the secondary outcome was referral to hospice. WLST and hospice referral information are collected by trained TQP personnel based on physicians’ orders, discharge instructions, nursing flow sheets, case manager/social workers’ notes, and discharge summaries. WLST is defined by TQP as removing or withholding life support interventions, such as ventilator support, renal replacement therapy, vasoactive medications, and invasive procedures (e.g., surgical or radiological procedures) [15].

### Statistical Analysis

Patients were stratified by race and ethnicity group, and their baseline characteristics, severity of brain injury, and hospital complications were summarized as the median value and interquartile range (Quartile 1, Quartile 3) or the number and percentage of patients. Variables were then compared between groups using either the Kruskal–Wallis test or the Chi-squared Test. To calculate risk-adjusted odds of WLST or utilization of hospice for patients of different races/ethnicities, we fitted multivariable logistic regression models. For the models, race and ethnicity category was included as an independent variable along with covariates that were selected a priori and were believed to be associated with the exposure, outcome, or both the exposure and outcome [16]. The dependent variable in the models was either WLST or utilization of hospice. For variables in the model, we reported adjusted odds ratios with 95% confidence intervals. Model fit was evaluated using the Hosmer–Lemeshow test. For all tests, *p* values < 0.05 were considered statistically significant. The manuscript was referenced against the Strengthening the Reporting of Observational Studies in Epidemiology (STROBE) checklist.

## Results

**Fig. 1:**
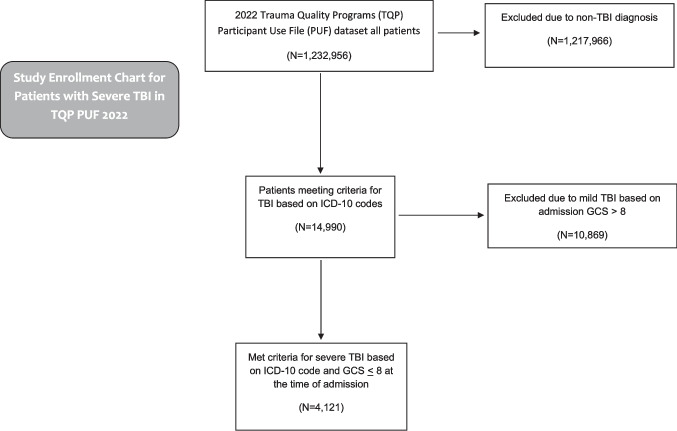
The study enrollment diagram

Figure 1 shows the study enrollment diagram. There were 4121 patients with severe TBI who were included in the final study cohort. Among these patients, 57.4% were non-Hispanic White, 17.3% were non-Hispanic Black, 14.3% were Hispanic, and 11.0% were non-Hispanic patients of other races. Table 1 lists patient demographics, comorbidities, initial vital signs, and markers of TBI severity. Non-Hispanic White patients with severe TBI were significantly older; median age = 51 years vs. 33 years in non-Hispanic Black patients and 34 years in Hispanic patients. Non-Hispanic patients of other races had a median age of 44 years (*p* value for four-group comparison < 0.001). The median GCS on arrival was 3 in all four race/ethnicity groups, and the highest GCS during hospitalization was 6 in all four groups. The percentage of patients with non-reactive pupils on arrival was highest in non-Hispanic Black patients at 48.6%, and lowest in Hispanic patients at 35.7% (*p* < 0.001 for four-group comparison). Midline shift on initial imaging, within 24 h of admission, varied between 26.3% for non-Hispanic Black patients and 34.8% for non-Hispanic patients of other races and was significantly different between the four groups (*p* < 0.001 for four-group comparison). Injury severity score varied between 26 and 27 and was highest in non-Hispanic Black patients (*p* = 0.02).

**Table 1:** Characteristics of patients with severe traumatic brain injury

Variable	Non-Hispanic White *N* = 2364	Non-Hispanic Black *N* = 712	Hispanic *N* = 588	Non-Hispanic other races *N* = 457	*p* value
Age (years)	51 [33, 69]	33 [25, 50]	34 [24, 49]	44 [28, 63]	<.001
Sex (% male)	1709 (72.3)	545 (76.7)	471 (80.1)	345 (75.7)	<.001
Body mass index (kg/m^2^)	26.5 [23.1, 30.3]	25.7 [22.1, 30.5]	26.6 [23.8, 30.2]	25.9 [22.7, 29.4]	.02
Insurance type					<.001
Medicaid	365 (15.4)	222 (31.2)	172 (29.2)	101 (22.1)	
Self-pay	319 (13.5)	174 (24.4)	144 (24.5)	99 (21.7)	
Commercial	816 (34.5)	180 (25.3)	165 (28.1)	134 (29.3)	
Medicare	662 (28.0)	71 (10.0)	56 (9.5)	78 (17.1)	
Other	202 (8.6)	65 (9.1)	51 (8.7)	45 (9.8)	
Hospital type*					<.001
For-profit	282 (11.9)	78 (11.0)	95 (16.2)	81 (17.7)	
Not-for-profit	2046 (86.6)	615 (86.5)	483 (82.1)	372 (81.4)	
Government	35 (1.5)	18 (2.5)	10 (1.7)	4 (0.9)	
Hospital size (beds)					<.001
200 or fewer	157 (6.6)	32 (4.5)	32 (5.4)	31 (6.8)	
201 to 400	648 (27.4)	134 (18.8)	155 (26.4)	132 (28.9)	
401 to 600	617 (26.1)	197 (27.7)	189 (32.1)	116 (25.3)	
More than 600	942 (39.9)	349 (49.0)	212 (36.1)	178 (39.0)	
Initial systolic blood pressure (mmHg)	130 [107, 157]	125 [94, 150]	130 [103, 150]	129 [102, 152]	<.001
Initial pulse rate (beats per minute)	92 [74, 113]	93 [69, 117]	98 [79, 119]	92 [71, 113]	.004
Initial temperature (°C)	36.3 [35.7, 36.7]	36.3 [35.7, 36.6]	36.4 [35.9, 36.7]	36.2 [35.6, 36.7]	.08
Initial oxygen saturation (%)	99 [95, 100]	99 [95, 100]	98 [95, 100]	98 [95, 100]	.21
Respiratory assistance at hospital arrival	632 (26.7)	200 (28.1)	209 (35.5)	135 (29.5)	<.001
Glasgow Coma Scale on arrival	3 [3, 5]	3 [3, 4]	3 [3, 6]	3 [3, 5]	<.001
Highest Glasgow Coma Scale	6 [3, 9]	6 [3, 8]	6 [3, 8]	6 [3, 8]	.03
Non-reactive pupils on admission	932 (39.4)	346 (48.6)	210 (35.7)	216 (47.3)	<.001
Midline shift	779 (33.0)	187 (26.3)	166 (28.2)	159 (34.8)	<.001
Injury severity score	26 [21, 34]	27 [22, 35]	26 [21, 35]	26 [25, 34]	.02
Positive opioid screen	47 (2.0)	6 (0.8)	11 (1.9)	12 (2.6)	.09
Positive alcohol screen	508 (21.5)	162 (22.8)	169 (28.7)	99 (21.7)	.002
Existing advanced directive	106 (4.5)	6 (0.8)	3 (0.5)	10 (2.2)	<.001
Prior cerebral vascular accident	57 (2.4)	8 (1.1)	5 (0.8)	8 (1.8)	.03
Chronic obstructive pulmonary disease	119 (5.0)	8 (1.1)	1 (0.2)	10 (2.2)	<.001
End stage renal disease	20 (0.8)	7 ((0.9)	5 (0.9)	5 (1.1)	.005
Congestive heart failure	61 (2.6)	9 (1.3)	5 (0.9)	7 (1.5)	.02
Diabetes mellitus	227 (9.6)	49 (6.9)	45 (7.7)	38 (8.3)	.10

*2 patients missing data

Table 2 lists hospital events and in-hospital complications. Transfusion of both red blood cells (RBCs) and fresh frozen plasma (FFP) was highest in non-Hispanic Black patients (*p* value for four-group comparison < 0.001). Surgery for hemorrhage control was most common in Hispanic patients, 9.0%, and least common in non-Hispanic White patients, 4.8% (*p* value for four-group comparison < 0.001). The use of an intracranial pressure monitor did not differ significantly between groups and was between 23.3 and 27.7% of patients in the four groups (*p* = 0.16). Cardiac arrest was significantly more common in non-Hispanic White patients, as was acute kidney injury (*p* < 0.001), while all other complications were similar between the four groups.

**Table 2:** Hospital events in patients with severe traumatic brain injury

Variable	Non-Hispanic White *N* = 2364	Non-Hispanic Black *N* = 712	Hispanic *N* = 588	Non-Hispanic other races *N* = 457	*p* value
Packed RBCs transfused first 4 h	519 (22.0)	216 (30.3)	163 (27.7)	127 (27.8)	<.001
FFP transfused first 4 h	358 (15.1)	157 (22.1)	109 (18.5)	91 (19.9)	<.001
Platelets transfused first 4 h	217 (9.2)	72 (10.1)	66 (11.2)	54 (11.8)	.22
Surgery for hemorrhage control	114 (4.8)	62 (8.7)	53 (9.0)	30 (6.6)	<.001
Intracranial pressure monitor used	559 (23.7)	166 (23.3)	163 (27.7)	118 (25.8)	.16
Cardiac arrest	396 (18.0)	108 (17.0)	52 (10.0)	33 (8.4)	<.001
Deep surgical site infection	20 (0.9)	2 (0.3)	1 (0.2)	1 (0.3)	.12
Deep vein thrombosis	81 (3.4)	17 (2.4)	11 (1.9)	8 (1.8)	.07
Myocardial infarction	11 (0.5)	2 (0.3)	2 (0.3)	0 (0.0)	.62
Severe sepsis	1 (0.1)	0 (0.0)	0 (0.0)	1 (0.3)	.31
Pulmonary embolism	20 (0.9)	7 (1.0)	4 (0.7)	2 (0.4)	.79
Ventilator associated pneumonia	13 (0.6)	4 (0.6)	1 (0.2)	2 (0.5)	.70
Acute kidney injury stage 3 or greater	27 (1.2)	0 (0.0)	0 (0.0)	2 (0.5)	<.001

*FFP*, fresh frozen plasma; *RBC*, red blood cell

Table 3 lists data on the length of hospital and ICU stay as well as WLST and discharge location. The median ICU length of stay was between 5 and 8 days in the four groups and was longest in non-Hispanic Black patients (*p* value for four-group comparison < 0.001). The percentage of patients who had WLST differed significantly between groups and was highest in non-Hispanic White patients, 32.0%, and was lowest in Hispanic patients, 16.3% (*p* value for four-group comparison < 0.001). In-hospital mortality was highest for non-Hispanic White patients, 43.1%, and lowest for Hispanic patients, 33%. Referral to hospice was most common in non-Hispanic White patients, 5.3%, and least common in non-Hispanic Black patients, 1.4% (*p* value < 0.001 for comparison of discharge location between 4 groups).

**Table 3:** Length of stay and discharge disposition in patients with severe traumatic brain injury

Variable	Non-Hispanic White *N* = 2364	Non-Hispanic Black *N* = 712	Hispanic *N* = 588	Non-Hispanic other Races *N* = 457	*p* value
Intensive care unit length of stay (days)	6 [2, 13]	8 [3, 15]	7 [3, 16]	5 [2, 13]	<.001
Hospital length of stay (days)	6 [2, 18]	7 [2, 22]	9 [2, 24]	4 [2, 18]	<.001
Total ventilator time (days)	2 [1, 4]	2 [1, 4]	2 [1, 4]	2 [1, 3]	.06
Withdrawal of life-sustaining treatment (WLST)	756 (32.0)	102 (14.3)	96 (16.3)	112 (24.5)	<.001
Withdrawal of life-sustaining treatment day	2 [1, 4]	2 [1, 4]	2 [1, 4]	2 [1, 3]	.50
Hospital discharge disposition					<.001
Died	1018 (43.1)	261 (36.7)	197 (33.5)	206 (45.0)	
Discharged to hospice	126 (5.3)	10 (1.4)	17 (2.9)	10 (2.2)	
Discharged to skilled nursing facility	94 (4.0)	40 (5.6)	32 (5.4)	18 (3.9)	
Discharged to inpatient rehab	366 (15.5)	121 (17.0)	100 (17.0)	72 (15.8)	
Discharged to long-term care hospital	123 (5.2)	42 (5.9)	35 (6.0)	24 (5.3)	
Discharge to self-care	232 (9.8)	84 (11.8)	111 (18.9)	38 (8.3)	
Other	405 (17.1)	154 (21.6)	96 (16.3)	89 (19.5)	

Table 4 lists the results of the multivariable regression models. Race and ethnicity were independently associated with the odds of WLST. Specifically, non-Hispanic Black patients had lower risk-adjusted odds of WLST compared to non-Hispanic White patients; OR = 0.49 (95% CI = 0.37 to 0.65, *p* < 0.001). Similarly, both Hispanic patients and non-Hispanic patients of other races also had lower risk-adjusted odds of WLST; OR = 0.59 (95% CI = 0.44 to 0.78, *p* < 0.001) and OR = 0.67 (95% CI = 0.49 to 0.90, *p* = 0.009). Other variables that had a significant association with WLST included advanced age, ISS, non-reactive pupils at admission, best GCS during hospitalization, and midline shift on initial imaging (all *p* < 0.05). Race and ethnicity were also associated with hospice referral. Specifically, non-Hispanic Black patients had lower risk-adjusted odds of hospice referral; OR = 0.42 (95% CI = 0.20 to 0.88, *p* = 0.02). Male patients also had lower risk-adjusted odds of hospice referral; OR = 0.68 (95% CI = 0.48 to 0.98, *p* = 0.04) while older patients were more likely to be referred to hospice; OR = 1.05 per year (95% CI = 1.04 to 1.06, *p* < 0.001).

**Table 4:** Multivariable models for withdrawal of life-sustaining treatment and hospice referral

Variable	Odds ratio with 95% CI for WLST	*p* value	Odds ratio with 95% CI for hospice referral	*p* value
Race/ethnicity category				
Non-Hispanic White	Ref		Ref	
Non-Hispanic Black	0.49 (0.37 to 0.65)	<.001	0.42 (0.20 to 0.88)	.02
Hispanic	0.59 (0.44 to 0.78)	<.001	0.97 (0.54 to 1.72)	.90
Other races, non-Hispanic	0.67 (0.49 to 0.90)	.009	0.56 (0.29 to 1.10)	.09
Male sex	0.90 (0.73 to 1.10)	.29	0.68 (0.48 to 0.98)	.04
Age (per year)	1.04 (1.04 to 1.05)	<.001	1.05 (1.04 to 1.06)	<.001
Injury severity score (per one point increase)	1.03 (1.02 to 1.03)	<.001	1.00 (0.98 to 1.02)	.88
Pupils non-reactive at admission	1.59 (1.30 to 1.94)	<.001	1.00 (0.67 to 1.50)	.98
Best Glasgow Coma Scale	0.81 (0.78 to 0.84)	<.001	0.98 (0.92 to 1.04)	.42
Presence of midline shift	1.49 (1.24 to 1.80)	<.001	0.99 (0.69 to 1.44)	.97
Advanced directives present on admission	1.07 (0.64 to 1.78)	.80	0.83 (0.39 to 1.75)	.62

*WLST*, withdrawal of life-sustaining treatment

## Discussion

In an observational cohort study of 4121 patients with severe TBI from across the USA, in-hospital mortality was 40.8%. The overall rate of WLST in the cohort was 25.9% with significantly lower risk-adjusted odds of WLST among non-Hispanic Black patients, Hispanic patients, and non-Hispanic patients of other races compared to non-Hispanic White patients. There was a low overall rate of hospice referral in the cohort, 3.9%, with a significantly lower rate for non-Hispanic Black patients compared to non-Hispanic White patients. Specifically, non-Hispanic Black patients had 58% lower risk-adjusted odds of hospice referral compared to non-Hispanic White patients. Taken together, these findings suggest significant differences remain in how patients of different races and ethnicities receive end-of-life care after TBI. In addition, the low rate of hospice referral in the cohort may represent an opportunity to improve patient- and family-centered care at the end of life in patients with severe TBI.

Severe TBI occurs in approximately 5.8 million persons per year globally and is among the top causes of trauma-related death and disability [17]. In a prior study of approximately 400 patients from a single major trauma center in the USA, mortality was 38% at discharge, 43% at 3 months, and 50% at 6 months with only 22% of patients having a good outcome or moderate disability at discharge [18]. In this study, predictors of poor outcome included advanced age and low GCS, absence of a pupillary response, presence of a midline shift on brain imaging, and an intracranial pressure ≥ 15 mmHg [18]. In a second study of just over 500 patients, where patients were followed for up to 8 years after their initial brain injury, mortality was 52% by 8 years. Of the patients who were alive at 8 years, 20% were severely disabled, 46% were moderately disabled, and 34% had a good recovery [19].

Almost all trauma patients with severe TBI require surrogate decision-making, and only a small fraction of patients (10–15%) has an advance directive at the time of their injury [5, 20]. Given the difficulties of accurate prognostication in TBI [21, 22], surrogate decision makers are often left with difficult decisions and in some cases have limited medical literacy to help guide them. The American College of Surgeons (ACS) statement on best practices in TBI recognizes difficulty in accurate prognostication after TBI and also states that early transition to do not resuscitate (DNR) or WLST is associated with higher mortality [23]. For this reason, ACS recommends that all patients with severe TBI receive at least 72 h of aggressive treatment post-injury [23]. Notably, in our study, the median day where WLST occurred was on hospital day 2, which is before the 72-h mark recommended by ACS. Although this suggests that some patients did not receive the recommended duration of aggressive care, it is difficult to fully account for concurrent injuries, which may have worsened patients’ overall prognosis. A prior multicenter Canadian study found significant variability in WLST for patients with severe TBI with odds ratios for WLST between 0.4 and 2.4 for different centers [24]. In this study, half of deaths occurred within the first 3 days of hospital admission, and two-thirds of these were related to WLST [24]. The most common reasons for WLST in this study were low likelihood of survival (54%) and a prognosis that was not consistent with the patient’s wishes (34%) [24]. Taken together, our data and prior data suggest that early WLST in patients with severe TBI is relatively common in Canada and the USA.

Surrogate decision-making in the ICU, particularly end-of-life decision-making, is influenced by many factors including perceived prognosis, the decision maker’s medical literacy, culture, religion, socioeconomic status, stress experienced by the decision maker, and overall beliefs about healthcare and the healthcare system [25–27]. The American College of Critical Care Medicine and the American Thoracic Society recommend a shared decision-making model, which is based on collaborative discussion and decision-making between the care team and surrogate decision maker [28]. Shared decisions should consider the best available scientific evidence, as well as the patient’s values and beliefs [28]. A multi-disciplinary approach to complex care discussions is often helpful for surrogates to make the best possible decision for the patient [29]. However, not all hospitals provide such an approach. Globally, nationally, regionally, and even within individual hospitals, there is significant variation in end-of-life care and WLST with one systematic review finding a variation of between 5 and 67% in various publications [30]. This suggests a need to better quantify the differences that exist and to better understand potential explanations.

Differences in end-of-life decision-making among patients of different races and ethnicities in the USA have been described in multiple prior studies [26]. For example, non-White patients less frequently have a DNR order while in the hospital [31]. Also, WLST has been found to differ based on religion, race, and ethnicity [26]. In regard to severe TBI, there is limited data describing the association between race and WLST. In one multi-year study of patients with severe TBI from 2013 to 2015, the authors found that both Black patients and patients of other races were less likely to have WLST (OR = 0.66 and 0.83 respectively) compared to White patients [13]. Limitations of this study included a lack of control for ethnicity (Hispanic vs. non-Hispanic), no formal analysis of hospice utilization, and a lack of control for age and ISS in the authors’ multivariable model [13].

Our study’s findings have both similarities and differences compared to this prior study. First, we found lower odds of WLST in non-Hispanic Black patients compared to non-Hispanic White patients. Second, we found that Hispanic patients were less likely to terminate life-sustaining treatment compared to non-Hispanic White patients after controlling for confounders. Third, we found that non-Hispanic Black patients had significantly lower risk-adjusted odds of having hospice referral compared to non-Hispanic White patients after controlling for confounders. Finally, we found slightly higher in-hospital mortality (40.8% vs. 33.9%) and WLST (25.9% vs. 20.7%) for severe TBI patients compared to what was previously reported, even though ISS was similar. This suggests that there is a trend towards more patients with severe TBI having WLST and dying in the hospital in the USA. These differences could represent both changes in clinical practice and surrogate decision-making over time.

The rate of hospice referral in our study is slightly increased from what was previously reported by Williamson et al. (3.9% vs. 2.1%) but remains low overall. In our opinion, this represents a potential opportunity to improve patient and family-centered care in TBI. In a recent large study of Medicare claims, palliative care and hospice utilization remained lower in both surgical and trauma patients compared to medical patients [32]. In their concluding remarks, the authors stated that the presence of severe TBI might be useful in helping to determine which trauma patients and families would benefit from hospice care [32]. Benefits of hospice care in severely injured trauma patients with a poor prognosis include a focus on comfort and quality of life, structured family support, enhanced autonomy for families, and the benefit of being in a familiar and more comfortable setting during the dying process [33, 34]. It is possible that patients in our study did not have equal access to hospice services based on the hospital where they presented or the region they lived. Disparities in access to hospice services have been well described in prior studies, both within the USA and other countries, particularly for non-cancer patients, rural patients, and patients from underrepresented racial and ethnic groups [11, 35].

Our study has several limitations. First, although we controlled for ISS in our multivariable model, it is challenging to completely control for the severity of patients’ injury state and the full degree of their brain injury. These factors may have biased our analysis to some degree. Second, our analysis took place during the coronavirus disease 2019 pandemic, and this may have impacted decision-making, particularly during periods of ICU bed shortage. Third, our analysis represents trauma and neurocritical care practices within the USA and may not be generalizable to other countries. Fourth, we did not have information on who initiated WLST for each patient, their relationship to the patient, the criteria that were used for decision-making, and the type of palliative care support that was offered to individual patients and their families. Finally, there is the potential for some patients’ race and ethnicity to have been misclassified, which may have led to bias.

In summary, in a contemporary analysis of over 4100 patients with severe TBI in the USA, we found an in-hospital mortality rate of 40.8% and significant differences in end-of-life care between patients of different races and ethnicities. Odds of WLST and hospice referral differed by up to 50–60% between race and ethnicity groups, and overall hospice utilization remained low. Our study’s primary findings are consistent with prior studies in severe TBI patients, although in-hospital mortality and WLST appear to have increased somewhat compared to 7 to 9 years ago in the USA. Further studies are needed to understand surrogate decision-making in severe TBI, to better delineate prognosis, and to optimize patient-centered end-of-life care when appropriate.

## Data Availability

The data used in this study can be accessed through the American College of Surgeons Trauma Quality Program. https://www.facs.org/quality-programs/trauma/quality/tqp-participant-hub/

## References

[1] Roozenbeek B, Chiu YL, Lingsma HF, Gerber LM, Steyerberg EW, Ghajar J, Maas AI. Predicting 14-day mortality after severe traumatic brain injury: application of the IMPACT models in the brain trauma foundation TBI-trac(R) New York State database. J Neurotrauma. 2012;29:1306–12. 10.1089/neu.2011.1988. 22150207 10.1089/neu.2011.1988 PMC3335134

[2] Dawes AJ, Sacks GD, Cryer HG, Gruen JP, Preston C, Gorospe D, Cohen M, McArthur DL, Russell MM, Maggard-Gibbons M, Ko CY. Intracranial pressure monitoring and inpatient mortality in severe traumatic brain injury: A propensity score–matched analysis. J Trauma Acute Care Surg. 2015;78(3):492–502. 10.1097/TA.0000000000000559. 25710418 10.1097/TA.0000000000000559

[3] Whitnall L, McMillan TM, Murray GD, Teasdale GM. Disability in young people and adults after head injury: 5–7 year follow up of a prospective cohort study. J Neurol Neurosurg Psychiatry. 2006;77:640–5. 10.1136/jnnp.2005.078246. 16614025 10.1136/jnnp.2005.078246 PMC2117429

[4] Andelic N, Howe EI, Hellstrom T, Sanchez MF, Lu J, Lovstad M, Roe C. Disability and quality of life 20 years after traumatic brain injury. Brain Behav. 2018;8:e01018. 10.1002/brb3.1018. 29888869 10.1002/brb3.1018 PMC6043714

[5] Graw JA, Burchard R. Completion rates of advance directives in a trauma emergency room: association with age. Emerg Med Int. 2021;2021:5537599. 10.1155/2021/5537599. 33968449 10.1155/2021/5537599 PMC8081623

[6] Sutter R, Meyer-Zehnder B, Baumann SM, Marsch S, Pargger H. Advance directives in the neurocritically ill: a systematic review. Crit Care Med. 2020;48:1188–95. 10.1097/CCM.0000000000004388. 32697490 10.1097/CCM.0000000000004388

[7] Graham M. Precedent autonomy and surrogate decisionmaking after severe brain injury. Camb Q Healthc Ethics. 2020;29:511–26. 10.1017/S0963180120000286. 32892770 10.1017/S0963180120000286 PMC7525111

[8] Muehlschlegel S, Rajajee V, Wartenberg KE, Alexander SA, Busl KM, Creutzfeldt CJ, Fontaine GV, Hocker SE, Hwang DY, Kim KS, Madzar D, Mahanes D, Mainali S, Meixensberger J, Sakowitz OW, Varelas PN, Weimar C, Westermaier T. Guidelines for neuroprognostication in critically ill adults with moderate-severe traumatic brain injury. Neurocrit Care. 2024;40:448–76. 10.1007/s12028-023-01902-2. 38366277 10.1007/s12028-023-01902-2 PMC10959796

[9] Hornor MA, Byrne JP, Engelhardt KE, Nathens AB. Examining racial disparities in the time to withdrawal of life-sustaining treatment in trauma. J Trauma Acute Care Surg. 2018;84:590–7. 10.1097/TA.0000000000001775. 29261591 10.1097/TA.0000000000001775

[10] Barnato AE, Anthony DL, Skinner J, Gallagher PM, Fisher ES. Racial and ethnic differences in preferences for end-of-life treatment. J Gen Intern Med. 2009;24:695–701. 10.1007/s11606-009-0952-6. 19387750 10.1007/s11606-009-0952-6 PMC2686762

[11] Ornstein KA, Roth DL, Huang J, Levitan EB, Rhodes JD, Fabius CD, Safford MM, Sheehan OC. Evaluation of racial disparities in hospice use and end-of-life treatment intensity in the REGARDS cohort. JAMA Netw Open. 2020;3:e2014639. 10.1001/jamanetworkopen.2020.14639. 32833020 10.1001/jamanetworkopen.2020.14639 PMC7445597

[12] Williams BM, Schneider A, Gallaher J, Charles A. Racial and ethnic disparities in withdrawal of life-sustaining treatment after non-head injury trauma. Am J Surg. 2022;223:998–1003. 10.1016/j.amjsurg.2021.08.007. 34384589 10.1016/j.amjsurg.2021.08.007 PMC8818056

[13] Williamson T, Ryser MD, Ubel PA, Abdelgadir J, Spears CA, Liu B, Komisarow J, Lemmon ME, Elsamadicy A, Lad SP. Withdrawal of life-supporting treatment in severe traumatic brain injury. JAMA Surg. 2020;155:723–31. 10.1001/jamasurg.2020.1790. 32584926 10.1001/jamasurg.2020.1790 PMC7301301

[14] Breathett K, Spatz ES, Kramer DB, Essien UR, Wadhera RK, Peterson PN, Ho PM, Nallamothu BK. The Groundwater of racial and ethnic disparities research: a statement from circulation: cardiovascular quality and outcomes. Circ Cardiovasc Qual Outcomes. 2021;14:e007868. 10.1161/CIRCOUTCOMES.121.007868. 33567860 10.1161/CIRCOUTCOMES.121.007868 PMC7958971

[15] Hoit G, Wijeysundera DN, Hamad DM, Nauth A, Atrey A, Halai M, Walser E, Nikouline A, Nathens AB, Khoshbin A. Insurance type and withdrawal of life-sustaining therapy in critically injured trauma patients. JAMA Netw Open. 2024;7:e2421711. 10.1001/jamanetworkopen.2024.21711. 39046743 10.1001/jamanetworkopen.2024.21711 PMC11270131

[16] VanderWeele TJ. Principles of confounder selection. Eur J Epidemiol. 2019;34:211–9. 10.1007/s10654-019-00494-6. 30840181 10.1007/s10654-019-00494-6 PMC6447501

[17] Iaccarino C, Carretta A, Nicolosi F, Morselli C. Epidemiology of severe traumatic brain injury. J Neurosurg Sci. 2018;62(5):535–41. 10.23736/S0390-5616.18.04532-0. 30182649 10.23736/S0390-5616.18.04532-0

[18] Schreiber MA, Aoki N, Scott BG, Beck JR. Determinants of mortality in patients with severe blunt head injury. Arch Surg. 2002;137:285–90. 10.1001/archsurg.137.3.285. 11888450 10.1001/archsurg.137.3.285

[19] Ruet A, Bayen E, Jourdan C, Ghout I, Meaude L, Lalanne A, Pradat-Diehl P, Nelson G, Charanton J, Aegerter P, Vallat-Azouvi C, Azouvi P. A Detailed overview of long-term outcomes in severe traumatic brain injury eight years post-injury. Front Neurol. 2019;10:120. 10.3389/fneur.2019.00120. 30846966 10.3389/fneur.2019.00120 PMC6393327

[20] Lee JS, Khan AD, Dorlac WC, Dunn J, McIntyre RC Jr, Wright FL, Platnick KB, Brockman V, Vega SA, Cofran JM, Duero C, Schroeppel TJ. The patient’s voice matters: The impact of advance directives on elderly trauma patients. J Trauma Acute Care Surg. 2022;92:339–46. 10.1097/TA.0000000000003400. 34538829 10.1097/TA.0000000000003400

[21] Maas AIR, Menon DK, Manley GT, Abrams M, Akerlund C, Andelic N, Aries M, Bashford T, Bell MJ, Bodien YG, Brett BL, Buki A, Chesnut RM, Citerio G, Clark D, Clasby B, Cooper DJ, Czeiter E, Czosnyka M, Dams-O'Connor K, De Keyser V, Diaz-Arrastia R, Ercole A, van Essen TA, Falvey E, Ferguson AR, Figaji A, Fitzgerald M, Foreman B, Gantner D, Gao G, Giacino J, Gravesteijn B, Guiza F, Gupta D, Gurnell M, Haagsma JA, Hammond FM, Hawryluk G, Hutchinson P, van der Jagt M, Jain S, Jain S, Jiang JY, Kent H, Kolias A, Kompanje EJO, Lecky F, Lingsma HF, Maegele M, Majdan M, Markowitz A, McCrea M, Meyfroidt G, Mikolic A, Mondello S, Mukherjee P, Nelson D, Nelson LD, Newcombe V, Okonkwo D, Oresic M, Peul W, Pisica D, Polinder S, Ponsford J, Puybasset L, Raj R, Robba C, Roe C, Rosand J, Schueler P, Sharp DJ, Smielewski P, Stein MB, von Steinbuchel N, Stewart W, Steyerberg EW, Stocchetti N, Temkin N, Tenovuo O, Theadom A, Thomas I, Espin AT, Turgeon AF, Unterberg A, Van Praag D, van Veen E, Verheyden J, Vyvere TV, Wang KKW, Wiegers EJA, Williams WH, Wilson L, Wisniewski SR, Younsi A, Yue JK, Yuh EL, Zeiler FA, Zeldovich M, Zemek R, In TP, Investigators. Traumatic brain injury: progress and challenges in prevention, clinical care, and research. Lancet Neurol. 2022, 21:1004–1060. 10.1016/S1474-4422(22)00309-X 10.1016/S1474-4422(22)00309-X PMC10427240 36183712

[22] Lazaridis C. Withdrawal of life-sustaining treatments in perceived devastating brain injury: the key role of uncertainty. Neurocrit Care. 2019;30:33–41. 10.1007/s12028-018-0595-8. 30143963 10.1007/s12028-018-0595-8

[23] ACS TQIP best practices in the management of traumatic brain injury. (2015). Accessed: November 15th, 2024: https://www.facs.org/media/mkej5u3b/tbi_guidelines.pdf.

[24] Turgeon AF, Lauzier F, Simard JF, Scales DC, Burns KE, Moore L, Zygun DA, Bernard F, Meade MO, Dung TC, Ratnapalan M. Mortality associated with withdrawal of life-sustaining therapy for patients with severe traumatic brain injury: a Canadian multicentre cohort study. Cmaj. 2011;183(14):1581–8. 10.1503/cmaj.101786. 21876014 10.1503/cmaj.101786 PMC3185074

[25] Iverson E, Celious A, Kennedy CR, Shehane E, Eastman A, Warren V, Freeman BD. Factors affecting stress experienced by surrogate decision makers for critically ill patients: implications for nursing practice. Intensive Crit Care Nurs. 2014;30:77–85. 10.1016/j.iccn.2013.08.008. 24211047 10.1016/j.iccn.2013.08.008 PMC3946606

[26] Frost DW, Cook DJ, Heyland DK, Fowler RA. Patient and healthcare professional factors influencing end-of-life decision-making during critical illness: a systematic review. Crit Care Med. 2011;39:1174–89. 10.1097/CCM.0b013e31820eacf2. 21336127 10.1097/CCM.0b013e31820eacf2

[27] Gopalan PD, Pershad S. Decision-making in ICU - a systematic review of factors considered important by ICU clinician decision makers with regard to ICU triage decisions. J Crit Care. 2019;50:99–110. 10.1016/j.jcrc.2018.11.027. 30502690 10.1016/j.jcrc.2018.11.027

[28] Kon AA, Davidson JE, Morrison W, Danis M, White DB. Shared decision-making in intensive care units executive summary of the american college of critical care medicine and american thoracic society policy statement. Am J Respirator Crit Care Med. 2016;193(12):1334–6. 10.1164/rccm.201602-0269ED. 10.1164/rccm.201602-0269ED PMC4910896 27097019

[29] Bhangu JK, Young BT, Posillico S, Ladhani HA, Zolin SJ, Claridge JA, Ho VP. Goals of care discussions for the imminently dying trauma patient. J Surg Res. 2020;246:269–73. 10.1016/j.jss.2019.07.046. 31614324 10.1016/j.jss.2019.07.046 PMC7006367

[30] Mark NM, Rayner SG, Lee NJ, Curtis JR. Global variability in withholding and withdrawal of life-sustaining treatment in the intensive care unit: a systematic review. Intensive Care Med. 2015;41:1572–85. 10.1007/s00134-015-3810-5. 25904183 10.1007/s00134-015-3810-5

[31] Shepardson LB, Youngner SJ, Speroff T, Rosenthal GE. Increased risk of death in patients with do-not-resuscitate orders. Med Care. 1999;37:727–37. 10.1097/00005650-199908000-00003. 10448716 10.1097/00005650-199908000-00003

[32] Fakhry SM, Carrick MM, Hoffman MR, Shen Y, Garland JM, Wyse RJ, Watts DD. Hospice and palliative care utilization in 16 004 232 medicare claims: comparing trauma to surgical and medical inpatients. Trauma Surg Acute Care Open. 2024;9:e001329. 10.1136/tsaco-2023-001329. 38646618 10.1136/tsaco-2023-001329 PMC11029464

[33] Wajid M, Rajkumar E, Romate J, George AJ, Lakshmi R, Simha S. Why is hospice care important? An exploration of its benefits for patients with terminal cancer. BMC Palliat Care. 2021;20:70. 10.1186/s12904-021-00757-8. 34001076 10.1186/s12904-021-00757-8 PMC8130431

[34] Sheikh M, Sekaran S, Kochhar H, Khan AT, Gupta I, Mago A, Maskey U, Marzban S. Hospice vs palliative care: a comprehensive review for primary care physician. J Family Med Prim Care. 2022;11:4168–73. 10.4103/jfmpc.jfmpc_2262_21. 36352983 10.4103/jfmpc.jfmpc_2262_21 PMC9638637

[35] Tobin J, Rogers A, Winterburn I, Tullie S, Kalyanasundaram A, Kuhn I, Barclay S. Hospice care access inequalities: a systematic review and narrative synthesis. BMJ Support Palliat Care. 2022;12:142–51. 10.1136/bmjspcare-2020-002719. 33608254 10.1136/bmjspcare-2020-002719 PMC9125370

